# Biocontrol of multidrug resistant pathogens isolated from fish farms using silver nanoparticles combined with hydrogen peroxide insight to its modulatory effect

**DOI:** 10.1038/s41598-024-58349-4

**Published:** 2024-04-04

**Authors:** Mai F. Saad, Mona M. Elsayed, Mariam Khder, Ahmed S. Abdelaziz, Azza S. El-Demerdash

**Affiliations:** 1 Department of Veterinary Public Health, Faculty of Veterinary Medicine, Zagazig University, Zagazig, 44511 Egypt; 2 Department of Hygiene and Zoonoses, Faculty of Veterinary Medicine, Mansoura University, Mansoura, 35516 Egypt; 3 Department of Pharmacology, Faculty of Veterinary Medicine, Zagazig University, Zagazig, 44519 Egypt; 4 Laboratory of Biotechnology, Department of Microbiology, Agricultural Research Center (ARC), Animal Health Research Institute (AHRI), Zagazig, 44516 Egypt

**Keywords:** Anti-virulence property, Aquaculture related drug resistant pathogens, Fish farms, Gene expression, Silver nanoparticles, Microbiology, Molecular biology

## Abstract

This study was divided into two parts. The first part involved the isolation, and detection of the prevalence and antimicrobial resistance profile of *Aeromonas hydrophila, Pseudomonas aeruginosa*, and *Vibrio* species from Nile tilapia fish and marine aquatic water. One hundred freshly dead Nile tilapia fish were collected from freshwater aquaculture fish farms located in Al-Abbassah district, Sharkia Governorate, and 100 samples of marine aquatic water were collected from fish farms in Port Said. The second part of the study focused on determining the in vitro inhibitory effect of dual-combination of AgNPs-H2O2 on bacterial growth and its down regulatory effect on crucial virulence factors using RT-PCR. The highest levels of *A. hydrophila* and* P. aeruginosa* were detected in 43%, and 34% of Nile tilapia fish samples, respectively. Meanwhile, the highest level of *Vibrio* species was found in 37% of marine water samples. Additionally, most of the isolated *A. hydrophila, P. aeruginosa* and *Vibrio* species exhibited a multi-drug resistance profile. The MIC and MBC results indicated a bactericidal effect of AgNPs-H2O2. Furthermore, a transcriptional modulation effect of AgNPs-H2O2 on the virulence-associated genes resulted in a significant down-regulation of *aerA, exoU,* and* trh* genes in *A. hydrophila, P. aeruginosa,* and* Vibrio spp*., respectively. The findings of this study suggest the effectiveness of AgNPs-H2O2 against drug resistant pathogens related to aquaculture.

## Introduction

The aquaculture industry has been growing rapidly in recent years to meet the global demand for seafood. Unfortunately, it is also prone to fish and shell fish related diseases caused by pathogenic microorganisms resulting in significant economic losses^1^. Water is a precious resource, and its usage in aquaculture places a heavy burden on it. Unconsumed food excretory byproducts, chemicals, and antibiotics can all lead to bacterial, fungal, and viral illnesses in the ponds. The typical remedy for this is constant water changes, which not only consumes hundreds of cubic meters of water per day (depending on the size of the pond) but also pollutes the surrounding environment with dirty effluent^2,3^. Of particular importance in aquaculture are the bacteria *Pseudomonas* species; *Aeromonas* species and *Vibrio* species, which can cause disease in fish^4^. One of the most virulent *Aeromonas* species; *A. hydrophila*, is capable of causing significant issues for Egypt's freshwater fish farming^5^. *Pseudomonas aeruginosa*, a gram-negative, rod-shaped, obligate aerobic bacterium, can infect fish, particularly in harsh environmental conditions, leading to ulcer-type disorders. Furthermore, *Pseudomonas aeruginosa*, the primary cause of nosocomial infections, can be fatal in immunocompromised individuals^6^. *Vibrio* species are ubiquitous foodborne pathogens found in surface waters and are associated with food poisoning^7^. They are abundant in estuarine-marine and freshwater ecosystems worldwide, often in association with aquatic creatures. Important *Vibrio* species include *V. parahaemolyticus, V. vulnificus, and V. alginolyticus*, which are primarily linked to waterborne illness^8^. Exposure to stressors such as pond water contamination can lead to a decrease in fish immunity and an increase in disease occurrence^9,10^. Bacterial resistance to conventional antibiotics is a serious problem that threatens the lives of people around the globe and imposes a significant economic burden on the health sector^11–16^. It is therefore inevitable to not only discover new antibiotics but also develop new non-antibiotic alternative approaches^17–20^. While methods are available for treating fish and shell fish-related diseases, they only offer a temporary solution and there is evidence of resistance to commercially available antibiotics. Therefore, there is an urgent need to find a reliable and effective environmentally-friendly alternative to conventional antibiotics to overcome the widespread issue of multidrug resistance (MDR) and its rapid emergence^21^. Nanotechnology, provides an excellent solution and has been proven effective for addressing three major concerns, water treatment, disease control and aquatic nutrients^1,22^. The use of nanomaterials particularly silver nanoparticles (AgNPs) has drawn interest from the academic, business, and nano medicine communities. A wide variety of Gram-positive and Gram-negative bacteria have been killed by AgNPs with outstanding bactericidal power^23^. Additionally, nanomaterials offer several benefits, including tissue-specific targeting, dosage and toxicity reduction, enhanced bioavailability, therapeutic effectiveness, and reduced secondary adverse effects^24,25^. The antimicrobial action of AgNPs also relies on reactive oxygen species (ROS) which induce cell death. Cell damage caused by ROS can manifest in various ways, such as disrupting protein synthesis within bacterial cells, inhibiting enzymes and damaging DNA and RNA^26,27^. Therefore, this study aimed to determine the prevalence rates of multidrug (MDR) pathogens in aquaculture including *A. hydrophila*, *P. aeruginosa,* and* Vibrio* spp. isolated from fish and farm water. Additionally, it was sought to assess the impact of commercially produced AgNPs-H2O2 on the expression of virulence genes in the isolated pathogens and evaluate its antibacterial efficacy on representative MDR isolates.

## Materials and methods

### Samples

Sampling was conducted in accordance with established guidelines^28^. A total of 100 freshly deceased Nile Tilapia fish were obtained from fresh water aquaculture fish farms in the Al-Abbassah district of Sharkia and promptly transferred to the bacteriology laboratory in an icebox. Additionally, 100 salt water samples were collected in labeled waterproof plastic bags from marine water fish farming in Port Said. This study was approved by the Research Ethics Committee of the Faculty of Veterinary Medicine, Zagazig University (Approval No ZU-IACUC/2/F/15/2023) and adhered to the ARRIVE guidelines (PLoS Bio 8(6), e1000412,2010).

### Bacteriological analysis

The water samples were concentrated on nitrocellulose membrane filters (0.45-μm Isolation of *A. hydrophila P. aeruginosa* and* Vibrio* spp. was conducted following a protocol previously established^5,29^. In summary, a loopful from the spleen, kidney, and liver of each collected fish was enriched in tryptic soy broth (TSB, Oxoid®, USA) tubes and then incubated at 37 °C for 18–24 h. To isolate typical yellow *A. hydrophila* colonies, a loopful was sub-cultured by streaking on Rimler-Shott’s medium (HiMedia, India) with novobiocin (Oxoid®, USA) supplement, and incubated for 24 h at 37 °C. For the isolation of typical fluorescent *P. aeruginosa* colonies, a loopful was sub-cultured by streaking on Pseudomonas F agar, and incubated for 24 h at 37 °C. The isolation of *Vibrio* spp. was carried out following a previously established protocol^30^.

The water samples were concentrated on nitrocellulose membrane filters (0.45-μm pore size; Millipore) by passing 100 mL through the filter using the membrane filtration technique. The filters were then placed onto alkaline peptone water (APW) and incubated for 24 h at 37 °C to enrich the growth of the *Vibrio* species. A loopful of culture from APW was streaked onto CHROMagar™ Vibrio (CHROMagar, Paris, France), and incubated at 37 °C for 24 h. Mauve, green–blue to turquoise blue, and creamy colonies representing *V. parahaemolyticus, V. vulnificus,* and* V. alginolyticus* respectively were picked up, and preserved.

### Molecular detection and genotypic characterization of *A. hydrophila P. aeruginosa * and *Vibrio* spp.

Five isolates of each microbe (*A. hydrophila, P. aeruginosa* and* Vibrio* spp.) were selected for molecular detection in the Biotechnology Unit, Animal Health Research Institute, Zagazig Branch, Egypt. Presumptive colonies were grown overnight on Tryptone Soy Broth (Oxoid, USA). Bacterial DNA was extracted using a QIAamp DNA Mini kit (Qiagen GmbH, Hilden, Germany) following the manufacturer’s instructions. Polymerase chain reaction (PCR) amplifications were performed using oligonucleotide primers specified in Table S1. These included the *aerA, act*, and *ast* genes of *A. hydrophila*; *toxA, exoS,* and* exoU* genes of *P. aeruginosa*, and *tdh, toxR, trh*, genes of *Vibrio* species along with *flaE, hsp,* and collagenase genes of *V. parahaemolyticus, V. vulnificus,* and* V. alginolyticus* respectively. The reference strains (control positive) used in this study were *Aeromonas hydrophila* ATCC® 35654™*, *Pseudomonas aeruginosa* ATCC® 27853™*, *Vibrio parahaemolyticus* ATCC® 17802™*, *Vibrio vulnificus* ATCC® 27562™*, and *Vibrio alginolyticus* ATCC® 17749™*.

### Antimicrobial susceptibility testing

The isolated A*. hydrophila, P. aeruginosa,* and* Vibrio* spp*.* were subjected to antibiotic sensitivity testing using the standard procedures for the disc diffusion method recommended by the Clinical Laboratory Standards Institute^31–34^ with selected panel of standard antimicrobial discs (Oxoid, Cambridge, UK). For *A. hydrophila*, the antibiotics tested included; ampicillin (AM, 10 μg), amoxicillin (AX, 25 μg), gentamicin (GM, 10 μg), colistin (CT, 10 μg), ciprofloxacin (CIP, 5 μg), erythromycin (E, 15 μg), doxycycline (DO, 30 μg), trimethoprim/sulfamethoxazole (SXT, 25 μg), and chloramphenicol (C, 30 μg). For *P. aeruginosa*, the antibiotics tested included gentamicin (GM, 10 μg), meropenem (MRP, 10 μg), ceftazidime (CAZ, 30 μg), ciprofloxacin (CIP, 5 μg), piperacillin-tazobactam (PTZ 100/10), aztreonam (ATM, 10 μg), fosfomycin (FF, 50 μg), and polymyxin B (PB, 300 μg). The antimicrobial susceptibility test for Vibrio species, included meropenem (MRP, 10 μg) ampicillin (AM, 10 μg), gentamicin (GM, 10 μg), ciprofloxacin (CIP, 5 μg), erythromycin (E, 15 μg), doxycycline (DO, 30 μg), and chloramphenicol (C, 30 μg). The multiple antimicrobial resistance indices were calculated as previously reported^35^. Pan drug-resistance (resistance to all antimicrobial agents), extensive drug-resistance (resistance to all classes of antimicrobial agents except 2 or fewer), and multidrug-resistance (resistance to three or more classes of antimicrobial agents) were determined as reported elsewhere^36^.

### Antimicrobial activities of AgNPs-H2O2

Bacterial strains that exhibited the highest levels of antimicrobial resistance profiles were chosen to test the antimicrobial effectiveness of AgNPs-H2O2 against them. These strains included five multidrug-resistant *A. hydrophila* strains, four extensively drug-resistant *P. aeruginosa* strains, and one pan drug-resistant *P. aeruginosa* strain, as well as five extensively drug-resistant *Vibrio* species. The Agar Well Diffusion Method was utilized to assess the antibacterial properties of AgNPs-H2O2 as follows:

Bacterial cultures were cultivated in sterile saline and adjusted to an optical density of 0.5 Macfarland (1.5 × 10^8^ CFU/mL). The bacterial suspension was evenly spread on Mueller Hinton (MH) agar (Oxoid Ltd., England) using a sterile cotton swab. Wells (8 mm) were created in each inoculated agar plate and approximately 10 µL of AgNPs-H2O2 at a concentration of 10% (v/v) was added into each well. Sterile water was used as a negative control for bacterial growth instead of AgNPs-H2O2. The agar plates were then placed in the incubator at 37 °C for 24 h. The experiments were conducted in triplicate, and the antimicrobial activity was determined by measuring the diameter of the inhibition zone (mm).

The effectiveness of AgNPs-H2O2 against the selected bacterial strains was evaluated using Minimum Inhibitory concentration (MIC) following the procedure described previously described^37^. The MIC was determined according to CLSI guidelines^38^ in a 96-well microtiter plate with 100 µL of a twofold serial dilution of commercial AgNPs-H2O2 in Muller Hinton broth (MHB) ranging from 32 to 0.0625 µg/mL, and bacterial suspension adjusted to 5 × 10^5^ CFU/mL pipetted into the wells. Sterile Muller Hinton broth without NPs served as a negative control, and inoculated Muller Hinton broth without NPs served as positive control. The microtiter plate was then incubated for 24 h at 37 °C. The MIC value was considered the lowest concentration of a particular antibacterial agent that could inhibit bacterial growth.

To determine the Minimum Bactericidal Concentration (MBC), 10 μL of bacterial suspension starting from the MIC onwards (equal to or two concentrations higher than the MIC) were dropped on MH agar and incubated as described above. The lowest concentration of each antimicrobial that killed 99.9% of bacterial growth was considered the MBC ^39^. Tolerance levels were determined using the formula: MBC/MIC ^40^, and the agent is considered bactericidal when the MBC/MIC ratio is ≤ 4^41^. The sub-inhibitory concentration was determined as the highest concentration of antimicrobial that showed no effect on survivability and no growth inhibition.

### Modulatory effect of AgNPs-H2O2

A reverse transcriptase quantitative PCR (RT-qPCR) analysis was conducted to investigate the modulatory effect of sub-inhibitory concentrations of AgNPs-H2O2, on the expression *of aerA, act,* and* ast* genes of *A. hydrophila, toxA, exoS,* and *exoU* genes of *P. aeruginosa,* and* tdh, toxR,* and *trh* genes for *Vibrio* species. Bacterial cultures were grown in the presence of AgNPs-H2O2 or without treatment as a reference control. Cells were harvested by centrifugation and cell pellet was mixed with 200 μL of RNA protect Bacteria Reagent (Qiagen, Hilden, Germany). Total RNA was extracted using the QIAamp RNeasy Mini kit (Qiagen, Germany, GmbH) following the manufacturer’s protocol. Residual DNA was removed by DNase digestion column using the RNase-Free DNase Set (Qiagen) protocol. RNA purity and concentration were determined using Nanodrop measurement. Real-time PCR amplification reactions were prepared in a final volume of 20 µL containing 10 µL of the 2× HERA SYBR® Green RT-qPCR Master Mix (Willowfort, UK), 1 µL of RT Enzyme Mix (20×), 1 µL of each primer at a concentration of 20 pmol, 2 µL of RNase- and DNase-free water, and 5 µL of RNA template. Quantitative Real-Time PCR (qPCR) was carried out using primers *for aerA, act,* and* ast* genes of* A. hydrophila*, *toxA*, *exoS,* and* exoU* genes of* P. aeruginosa*, and *tdh*, *toxR* and *trh*, genes of* Vibrio* species, and amplification conditions were set using a Real time PCR machine (StepOne Plus, Thermo Fisher). Results were analyzed using the comparative cycle threshold method (ΔΔCT method), and normalized against 16S rRNA as the endogenous control as previously described^42^.

### Statistical analysis

Each experiment was carried out at least in triplicate, and all data were presented as mean +/− standard deviation (SD). Analysis of statistical significance was performed by one-way analysis of variance (ANOVA) and the post hoc Tukey test (***p*** < 0.05). All analysis was conducted in SAS 9.4 for Windows 64-bit from SAS Institute (Cary, NC), and graphical outputs were generated by GraphPad Prism software (version 8; GraphPad Software Inc.).

## Results

### Prevalence of *A. hydrophila*, *P. aeruginosa, and Vibrio species*

**Figure 1:**
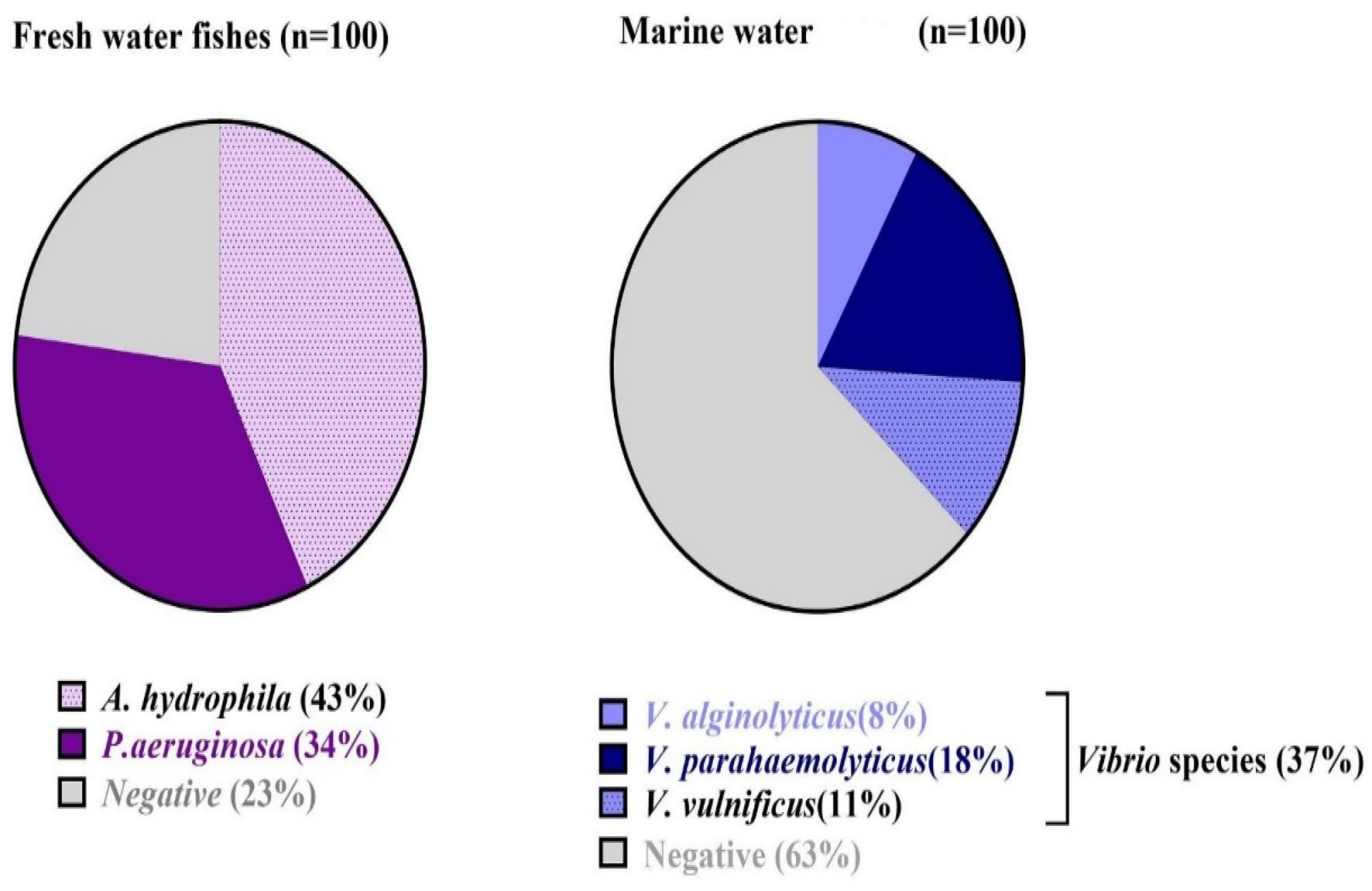
Prevalence of *A. hydrophila*, and *P. aeruginosa* species isolated from freshwater fishes, and *Vibrio* species from marine water including; *V. parahaemolyticus*, *V. vulnificus*, and *V. alginolyticus*.

The prevalence of *A. hydrophila* was 43% out of 100 samples of freshly deceased Nile tilapia (Fig. 1a, Table 1). Moreover *P. aeruginosa* was isolated with a percentage of 34% (Fig. 1a, Table 1). The overall higher prevalence of *A. hydrophila,* and *P. aeruginosa* was detected in liver samples (Tables S2 and S3). The isolation percentage of *Vibrio* species from marine water was 37% with *V. parahaemolyticus* isolates detected with a higher prevalence (18%) followed by *V. vulnificus*, and *V. alginolyticus* with 11% and 8% respectively (Fig. 1b, Table 1).

**Table 1:** Prevalence of *A. hydrophila,* and* P. aeruginosa* species isolated from freshwater fishes, and *Vibrio* species from marine water including; *V. parahaemolyticus, V. vulnificus,* and *V. alginolyticus.*.

Type of sample	*A. hydrophila*	*P. aeruginosa*	*Vibrio* species		
			*V. alginolyticus*	*V. parahaemolyticus*	*V. vulnificus*
Saltwater samples (n = 100)	0	0	18	8	11
Fresh water fishes (n = 100) Liver Kidney Spleen	17 14 12	14 11 9	0 0 0	0 0 0	0 0 0
Total (n = 200)	43	34	18	8	11

**Figure 2:**
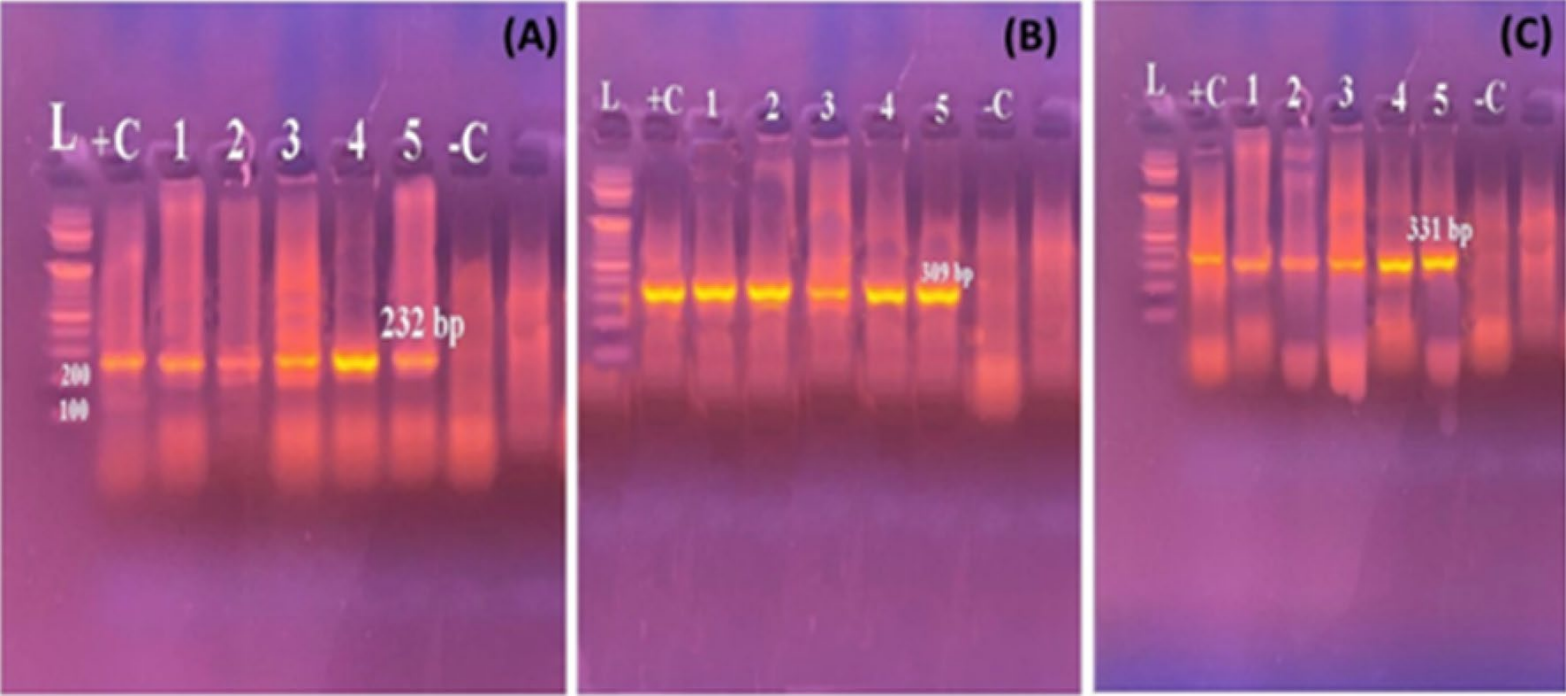
Representative agarose gel electrophoresis of *A. hydrophila* virulence genes showing PCR amplification for (**A**) *act* (232 bp), (**B**) *aerA* (309 bp), and (**C**) *ast* (331 bp) genes. Lane L, DNA ladder; Lane−C, negative controls; Lane + C, positive controls.

**Figure 3:**
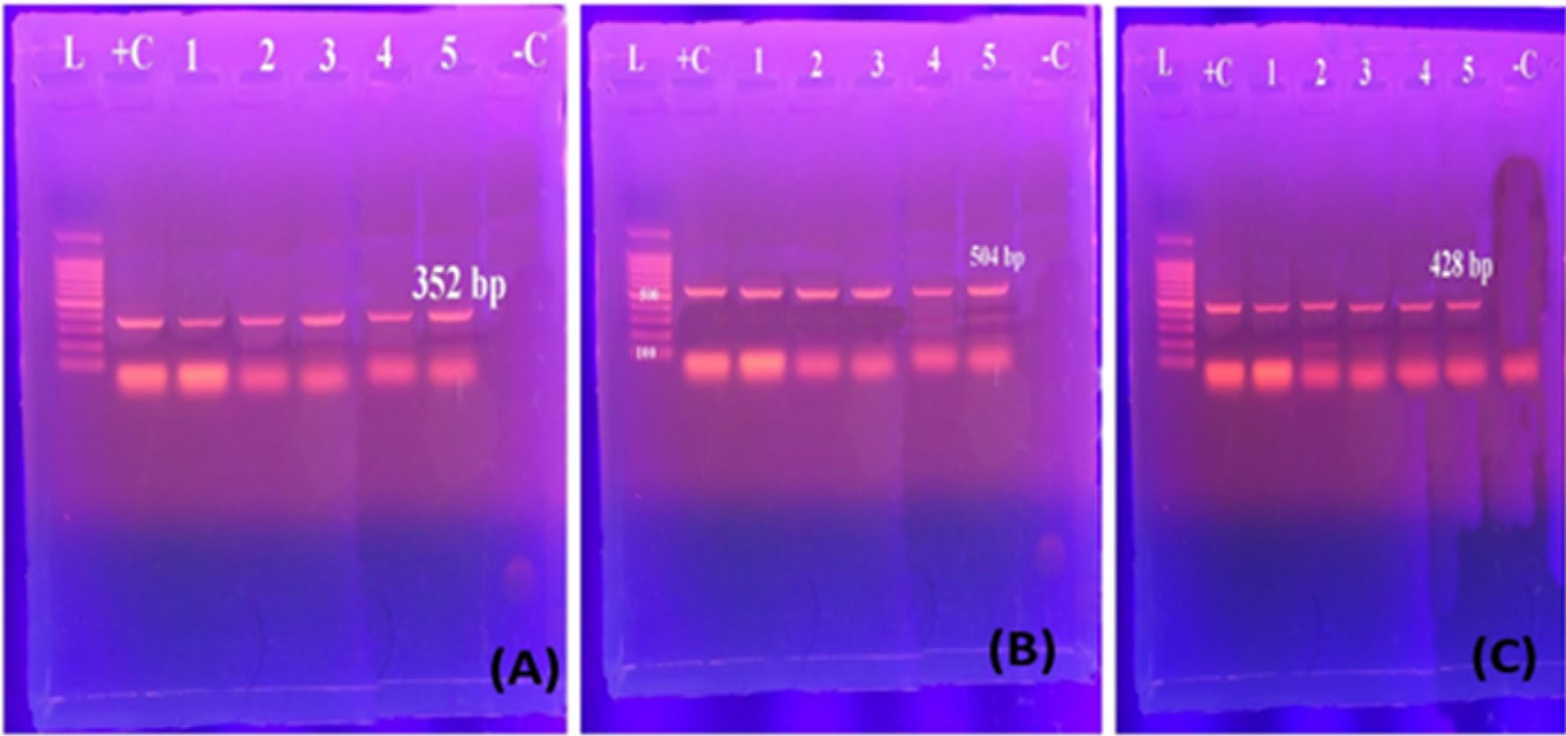
Representative agarose gel electrophoresis of *P. aeruginosa* showing PCR amplification for (**A**) *toxA* (352bp), (**B**) *exoS* (504bp), and (**C**) *exoU* (428bp) genes. Lane L, DNA ladder; Lane–C, negative controls; Lane + C, positive controls.

**Figure 4:**
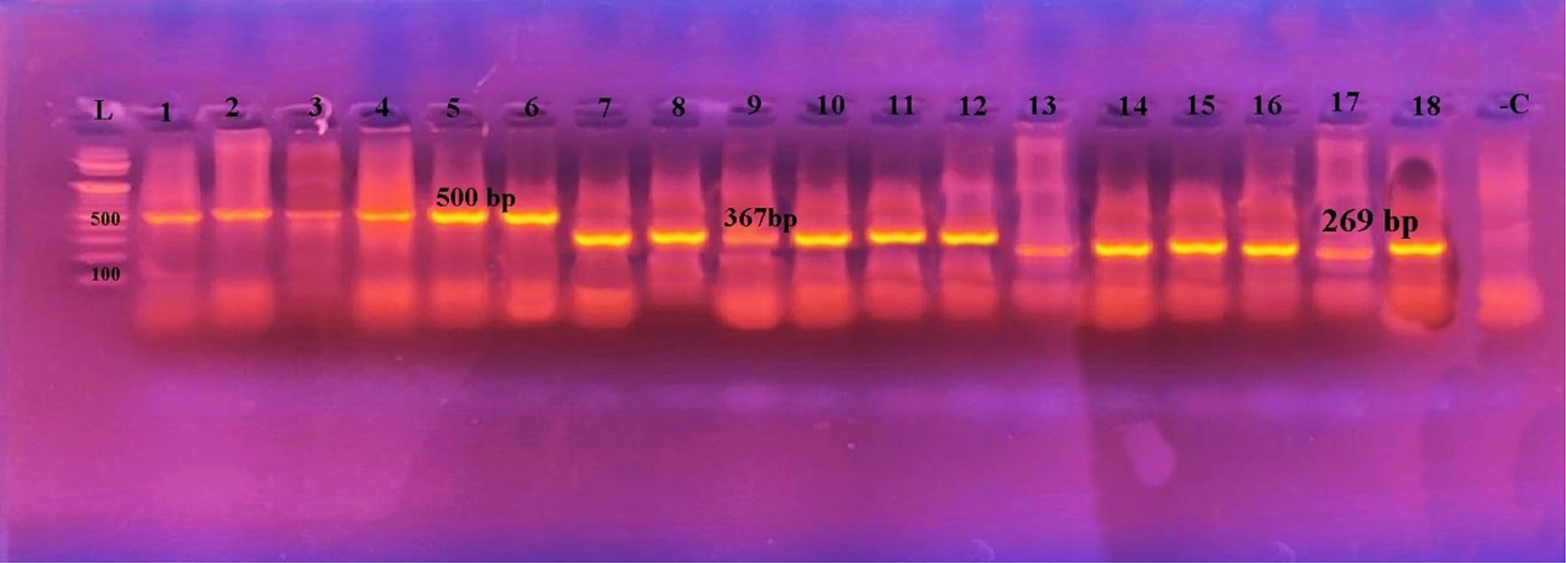
Agarose gel electrophoresis of five *Vibrio* isolates showing their genotypic patterns. Lane L: DNA ladder, Lanes 1, 7, 13: positive controls; Lanes 2–6: amplification pattern for *tdh* (500 bp), Lane 8–12: *toxR* (367 bp), Lanes 14–18: *trh* (269 bp) genes, and Lane −C: negative control.

**Figure 5:**
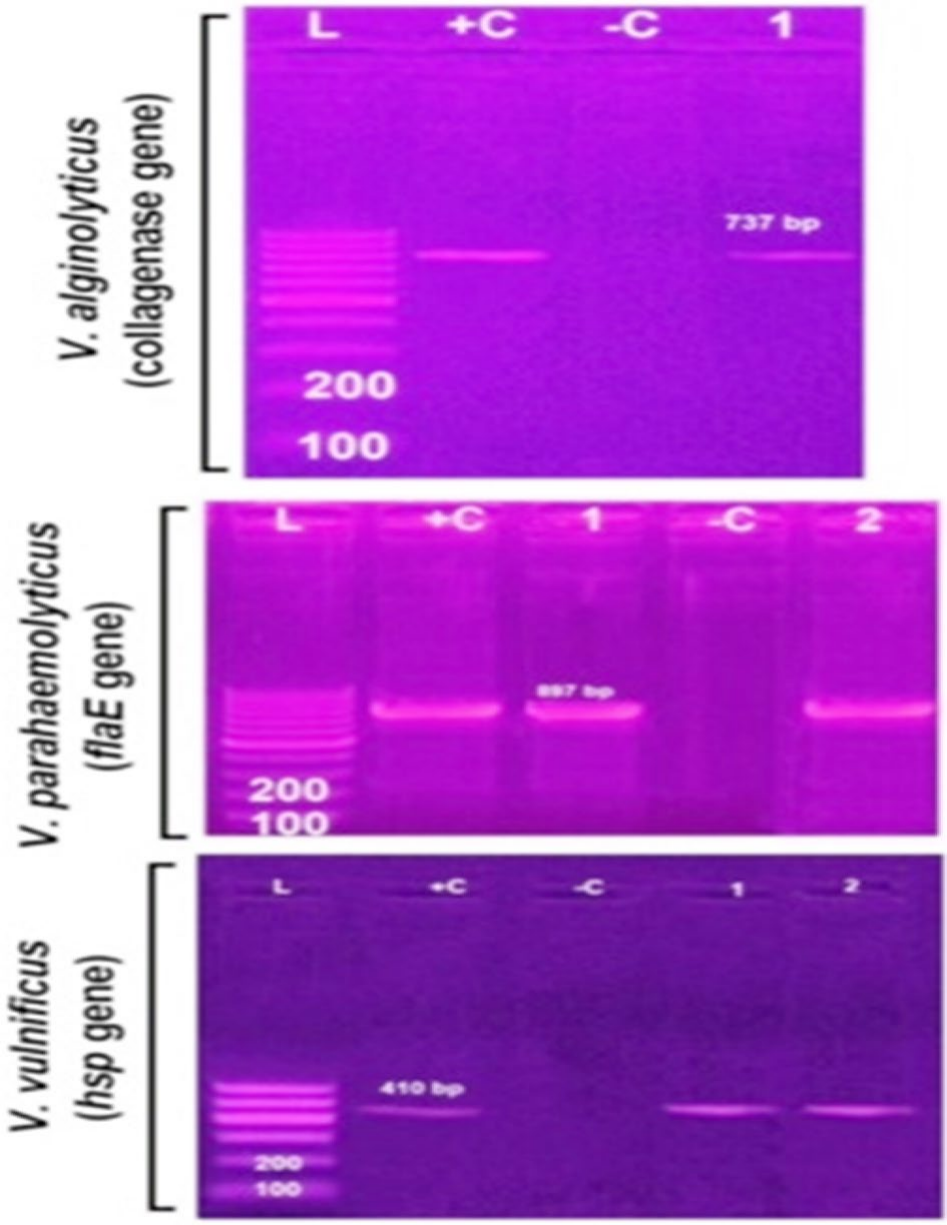
Agarose gel electrophoresis of *Vibrio* species showing PCR amplification of *flaE* gene (897 bp) of *V. parahaemolyticus*, *hsp* gene (410 bp) of *V. vulnificus*, and collagenase gene (737 bp) of *V. alginolyticus*. Lane L, DNA ladder; Lane −C, negative controls; Lane + C, positive controls.

Genotypic confirmation of *A. hydrophila* isolates showed PCR bands of 232bp, 309 bp, and 331bp harboring *act*, *aerA*, and *ast* genes, respectively (Fig. 2). PCR bands for *toxA*, *exoS*, and *exoU* genes of all examined *P. aeruginosa* (five isolates) showed 352bp, 504bp, and 428 bp, respectively (Fig. 3). Moreover, genotypic analysis for *Vibrio* species *tdh*, *toxR*, and trh harbored genes showed PCR bands of 500 bp, 367 bp, and 269 bp, respectively **(**Fig. 4**).** The presence of *flaE*, *hsp*, *collagenase* genes of *V. parahaemolyticus*, *V. vulnificus*, and *V. alginolyticus* respectively were shown in Fig. 5**.**

### Antimicrobial resistance profiles

**Figure 6:**
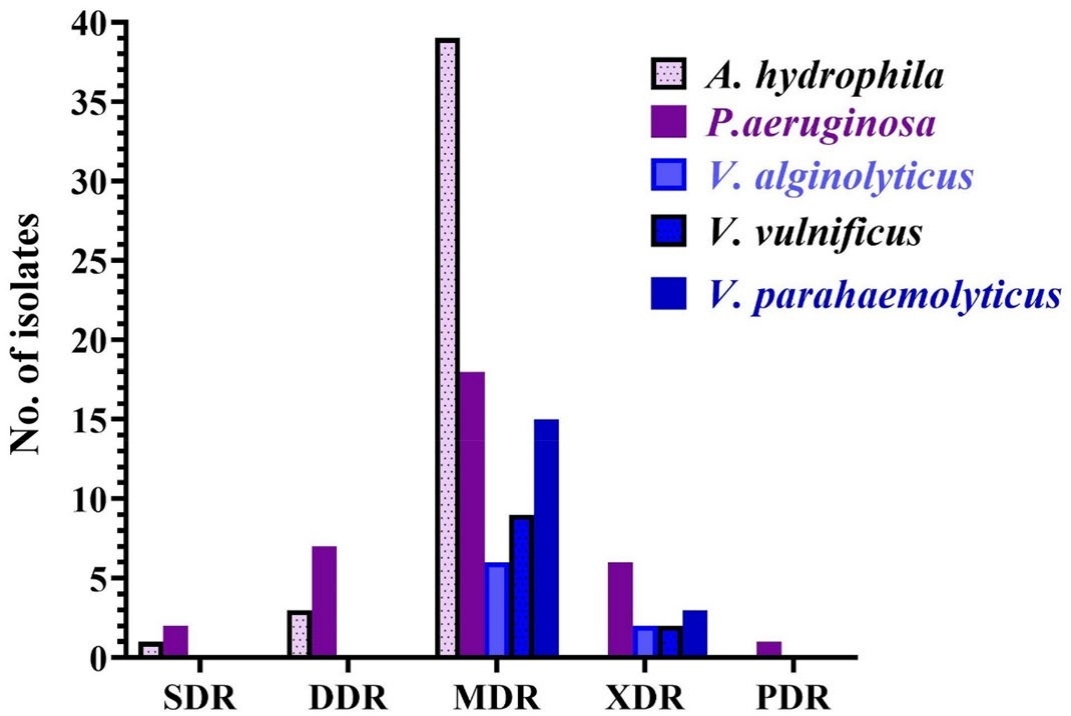
Occurrence of SDR (single drug resistant), DDR (double drug resistant), MDR (multidrug resistant), XDR (extensively drug resistant), and PDR (pan drug resistant) resistant patterns in *A. hydrophila, P. aeruginosa, V. parahaemolyticus, V. vulnificus,* and *V. alginolyticus*.

The in vitro antimicrobial susceptibilities of *A. hydrophila*, *P. aeruginosa,* and* Vibrio* species against antimicrobial agents are summarized in Tables S2, S3, and S4. The PDR, XDR, and MDR patterns were reported among the analyzed isolates (Fig. 6, Table 2). The MDR profiles were significantly increased among the *A. hydrophila* isolates with 90.69% (39 out of 43) exhibiting this resistant pattern. However, only 2% and 6%of the analyzed isolates showed SDR and DDR pattern respectively.

**Table 2:** Occurrence of SDR, DDR, MDR, XDR, and PDR categories in *A. hydrophila, P. aeruginosa, V. parahaemolyticus, V. vulnificus,* and *V. alginolyticus.*

Resistance category	Resistance to antimicrobial agent	Total no of antimicrobial agent	Resistant *bacterial species* (No. = number of bacteria)
SDR (n = 3)	1	9	*A. hydrophila* (1)
	1	8	*P. aeruginosa (2)*
DDR (n = 10)	2	9	*A. hydrophila* (3)
	2	8	*P. aeruginosa (7)*
MDR (*n* = 87)	3	9	*A. hydrophila* (10)
	3	8	*P. aeruginosa (9)*
	3	7	*V. alginolyticus (2), V. parahaemolyticus (6), V. vulnificus (4)*
	4	9	*A. hydrophila (14)*
	4	8	*P. aeruginosa (5)*
	4	7	*V. alginolyticus (4), V. parahaemolyticus (9), V. vulnificus (5)*
	5	9	*A. hydrophila* (7)
	5	8	*P. aeruginosa (4)*
	6	9	*A. hydrophila* (8)
XDR (*n* = 13)	6	8	*P. aeruginosa (2)*
	7	8	*P. aeruginosa (4)*
	5	7	*V. parahaemolyticus (1), V. vulnificus (1)*
	6	7	*V. alginolyticus (2), V. parahaemolyticus (2), V. vulnificus (1)*
PDR (*n* = 1)	8	8	*P. aeruginosa (1)*

In total, 18 isolates of *P. aeruginosa* exhibited MDR pattern (52.9%), 7 isolates exhibited XDR pattern (20.5%), and one isolate exhibited PDR pattern being resistant to all the tested antimicrobial agents (2.9%).

Considering *Vibrio* species, the multidrug resistant pattern was detected among most isolates (81%; 30 out of 37) with a higher prevalence of *V. parahaemolyticus* (50%;15 isolates) followed by *V. vulnificus*, and *V. alginolyticus* with 9 and 6 isolates (30%, and 20%) respectively. However, only 7 isolates showed an extensively drug resistant pattern (18.9%).

### Antibacterial effect of AgNPs-H_2_O_2_

**Figure 7:**
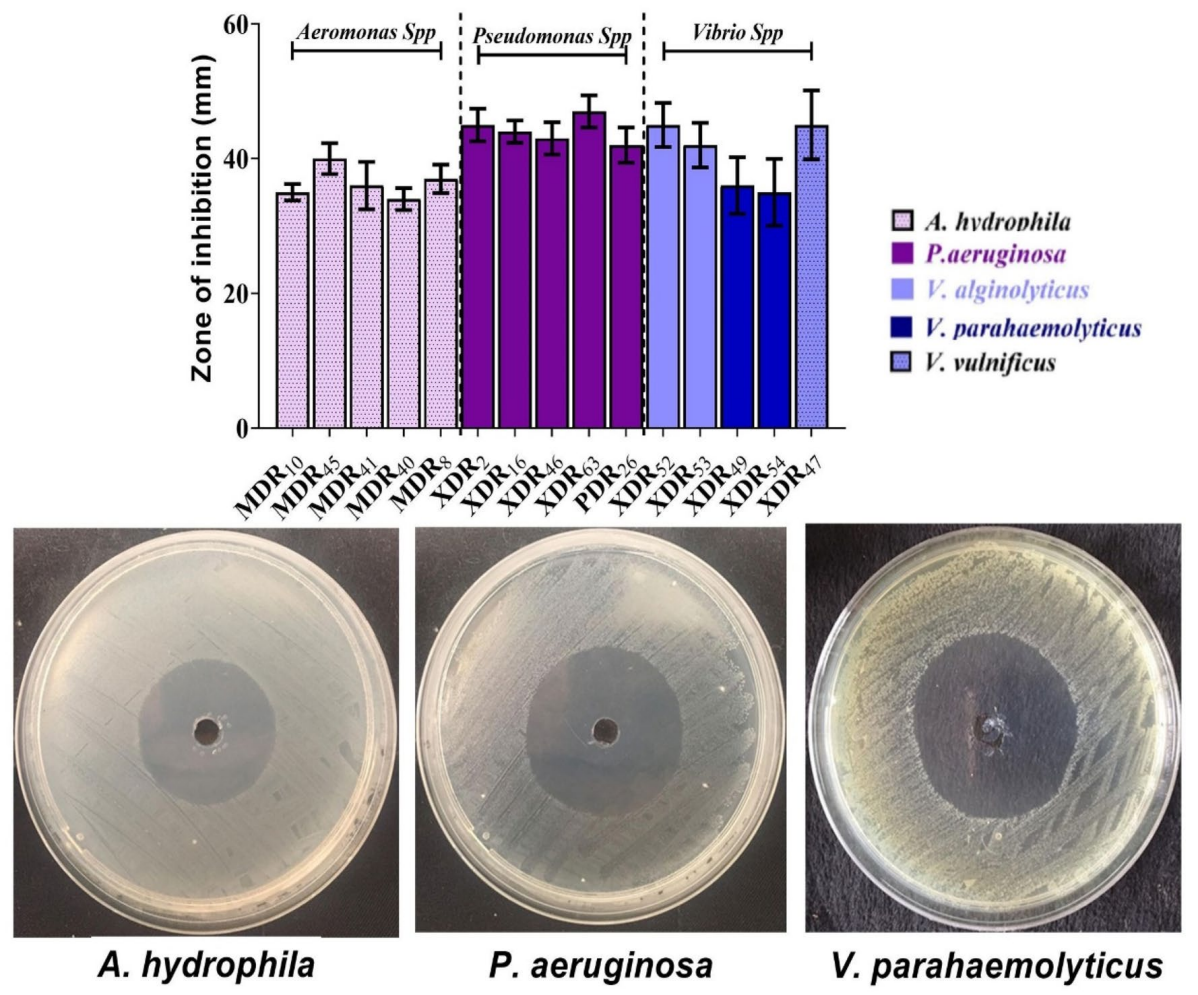
The inhibitory effect of AgNPs-H_2_O_2_ on *A. hydrophila, P. aeruginosa*, and *Vibrio* species growth. Antimicrobial susceptibility testing using the agar well diffusion method with representative images is shown in which AgNPs-H_2_O_2_ was used at concentration of 10% (v/v). The antibacterial activity was measured by the inhibition zone in millimeters (mm). The experiments were done in triplicate and each column represented the mean ± SEM.

Five *A. hydrophila* strains categorized as MDR (*n* = 5), five *P. aeruginosa* strains categorized as XDR (n = 4), and PDR (n = 1), and five XDR *Vibrio* species (n = 5) were selected and screened for the antibacterial properties of AgNPs-H_2_O_2_ using the agar well diffusion method, Minimum Inhibitory Concentration (MIC) and minimum bactericidal concentration (MBC). The inhibitory zones were presented in Fig. 7. A notable inhibition zone of 35–40 mm was observed with a 10% concentration of AgNPs-H_2_O_2_ against *A. hydrophila* strains, while the inhibition zone observed among tested *P. aeruginosa* was 42–47 mm. The inhibitory activity value ranged from 35 to 45 mm for vibrio species. Notably, the effect of AgNPs-H_2_O_2_against *Vibrio* species showed variable susceptibility as *V. parahaemolyticus* was less susceptible to AgNPs-H_2_O_2_ compared with *V. vulnificus*, and *V. alginolyticus*.

**Figure 8:**
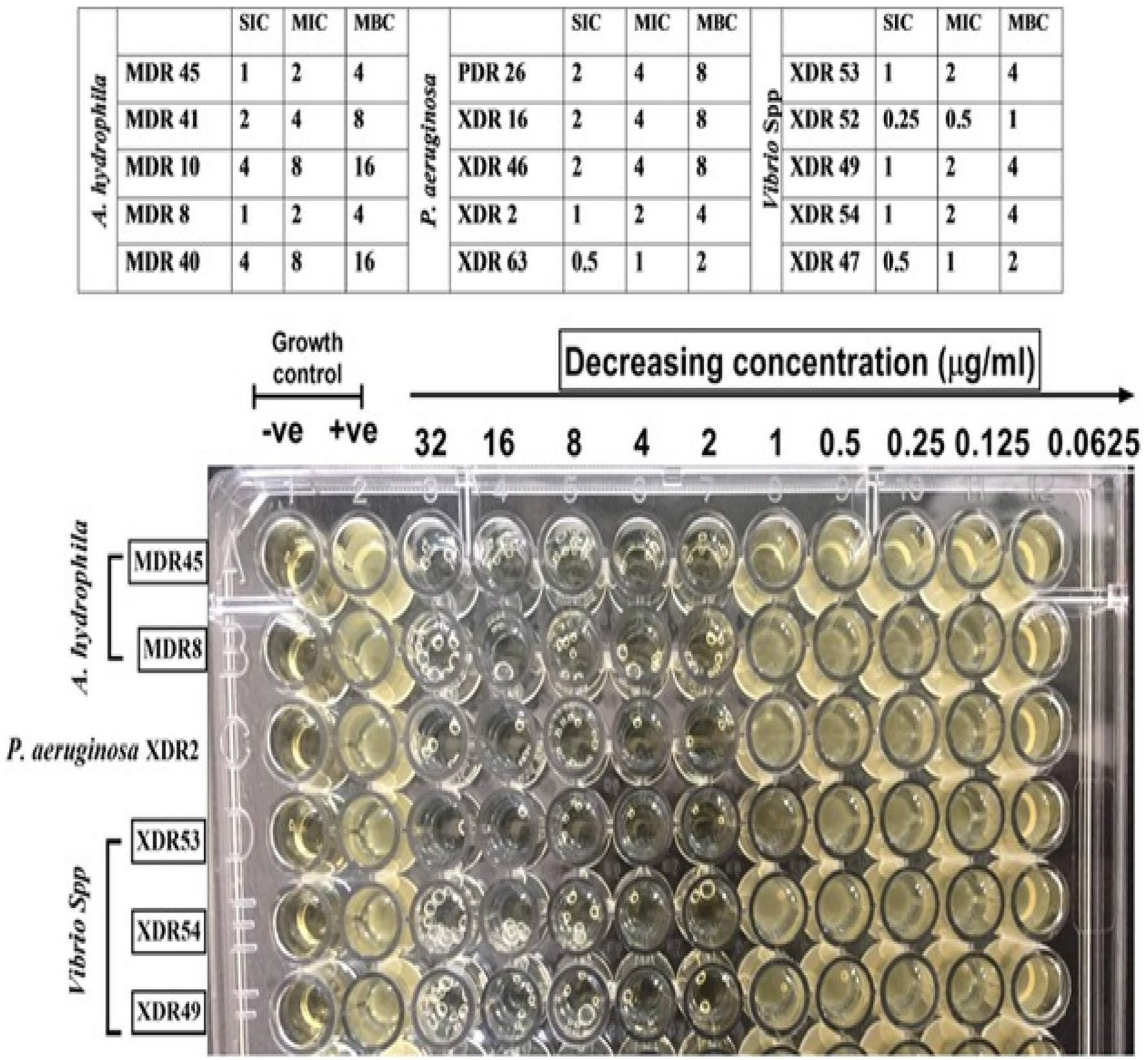
Minimum inhibitory concentration (MIC) of AgNPs-H_2_O_2_ among *A. hydrophila*, *P. aeruginosa*, and *Vibrio* species growth in compared with untreated positive control, and un-inoculated negative control. Antimicrobial susceptibility testing using the broth microdilution method with representative images are shown in which MIC was measured by 32–0.0625 μg/mL. SIC; sub-inhibitory concentration. MBC; minimum bactericidal concentration.

The Minimum Inhibitory Concentration (MIC), and Minimum Bactericidal Concentration (MBC) values were presented in Fig. 8. The MIC of all tested *A. hydrophila*, *P. aeruginosa, and Vibrio species* showed that AgNPs-H_2_O_2_ has an inhibitory effect on bacterial growth (0.5 to 8 µg/mL). The MIC values of AgNPs-H_2_O_2_ (2 µg/mL) were detected in 40% of the tested bacterial species including 2 strains of *A. hydrophila*, 3 strains of *Vibrio species* and 1 strain of *P. aeruginosa.* The MBC was noted at 4 µg/mL with a tolerance level of 2 indicating the bactericidal effect of AgNPs-H_2_O_2_. The MIC results illustrated that all tested *Vibrio* species were more susceptible to AgNPs-H_2_O_2_ than the other tested *A. hydrophila*, *and P. aeruginosa*, with a lower concentration value for inhibiting bacterial growth ranging from 0.5 to 2 µg/mL. These lower MIC values indicated greater antibacterial effectiveness.

### Transcriptional modulatory effect of AgNPs-H_2_O_2_

**Figure 9:**
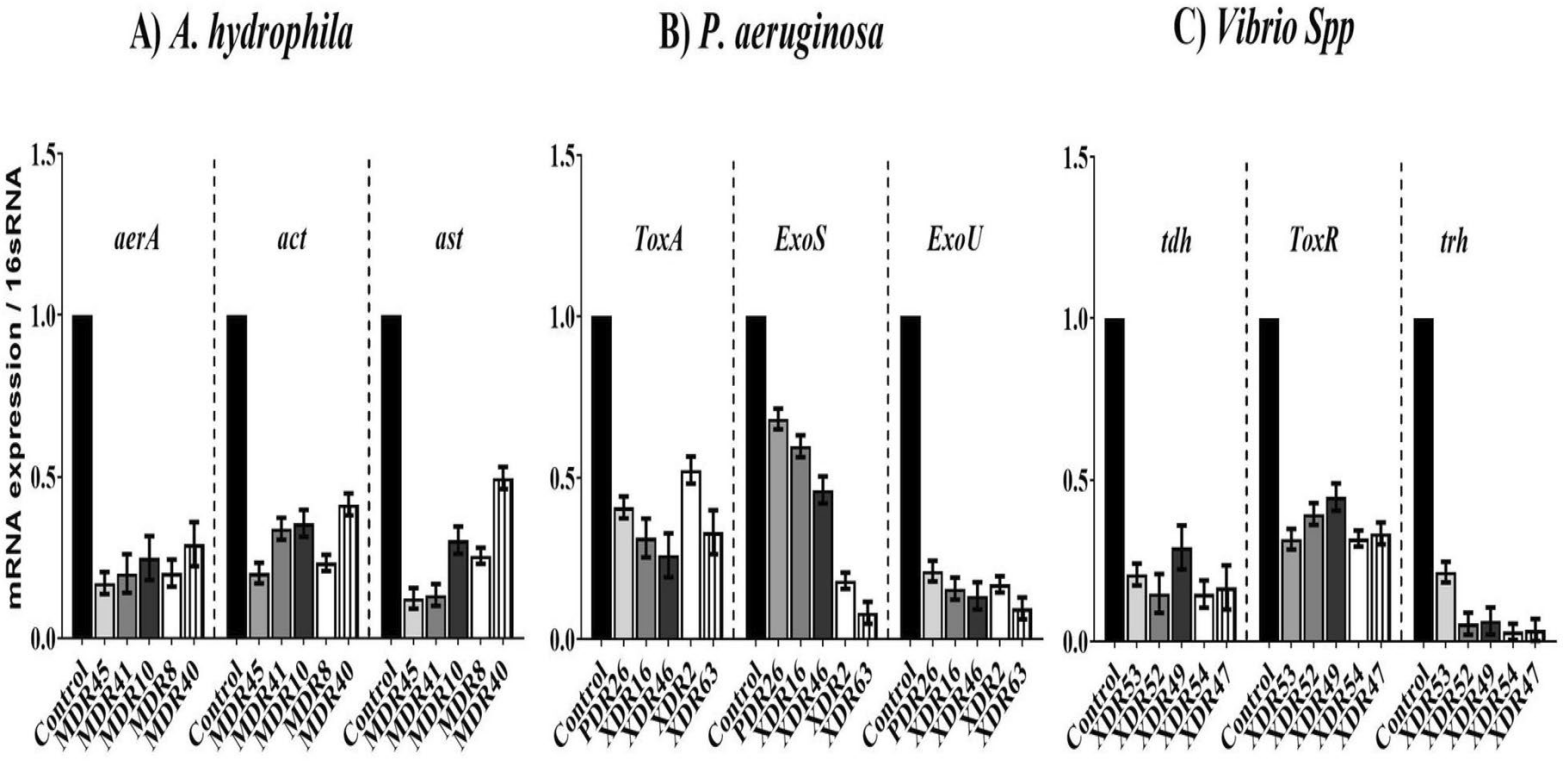
Relative expression of *A. hydrophilaact*, *aerA*, and *ast* genes (**A**), *P. aeruginosa, toxA*, *exoS*, and *exoU* genes (**B**), and Vibrio species *tdh*, *toxR*, and *trh* genes (**C**) upon treatment with AgNPs-H_2_O_2_. The fold change was determined by qRT-PCR, calculated using the ^ΔΔ^CT method and normalized comparatively to 16S rRNA expression. The experiments were done in triplicate and each column represented the mean ± SEM.

To investigate the mechanism by which AgNPs-H_2_O_2_ inhibit bacterial growth, real-time q RT-PCR was used to determine the expression levels of *act*, *aerA*, and *ast* genes in *5 multidrug resistant A. hydrophila* isolates, *toxA*, *exoS*, and *exoU* genes in *5 P. aeruginosa* isolates (4XDR, and 1PDR), and *tdh*, *toxR*, and *trh* in 5 extensively drug resistant *Vibrio* species reported here (Fig. 9). Data analysis indicated that all tested isolates showed down-regulation of the tested genes upon treatment with sub-inhibitory concentration of AgNPs-H_2_O_2_ compared to the untreated control isolates. The result indicated that *A. hydrophila* isolates treated with AgNPs-H_2_O_2_ had the most significant down-regulation of the *aerA* gene by 0.17–0.29-fold change. The suppression of *exoU* gene expression level among *P. aeruginosa* isolates was more pronounced with 0.09–0.21-fold change. Meanwhile, the *trh* gene expression level was the most affected among the tested vibrio species with a 0.029–0.21-fold change.

## Discussion

Aquaculture is essential for providing a reliable source of animal protein for human consumption in rapidly growing coastal nations like Egypt^43^. This supply is at risk from uncontrollable bacterial diseases, particularly from virulent and drug resistant pathogens such as *A. hydrophila, P. aeruginosa* and* Vibrio* spp. In this study, the prevalence of *A. hydrophila* in freshly deceased Nile tilapia was found to be 43%. Previous research has shown similar rates of *A. hydrophila* in Oreochromis niloticus^44–47^, while lower rates have also been reported^48–50^. The prevalence of *P. aeruginosa* in this study was consistent with previous findings with some studies reporting lower rates^51–53^. Previous studies have shown a higher prevalence of *V. parahaemolyticus* in farm water compared to this study^54–57^, while the isolation rate of *V. vulnificus* was similar to previous report^58^. The differences in prevalence rates of isolated bacteria could be due to factors such as the number of samples examined, variations in host susceptibility, geographical distribution, salt level, sampling period, and other environmental factors^59,60^.

Increasing resistance to major antibiotics is an emerging problem in pathogenic bacteria found in the aquatic environment including fish and pond water. Therefore, investigating of the drug resistance profiles is essential to prevent the spread of antibiotic-resistant pathogens and search for better alternatives. Multidrug-resistant (MDR) bacteria have become a global concern in recent decades posing a serious threat to human, animal and fish health^61^. In this study *A. hydrophila* isolates were MDR in 90.69% of cases. This level of resistance was lower than previously reported^62^, while a lower prevalence was also detected^63^. Meanwhile, the resistance profile of Pseudomonas isolates in this study showed a 52.9% MDR rates with a mean MAR index between 0.125 and 1 and an average of 0.48 similar to previous findings^64^. Regarding Vibrio isolates, 81% were found to be MDR, with most isolates displaying multiple MAR indices ranging from 0.42 to 0.86 and an average of 0.57. This suggests contamination from hazardous sources and acquired genetic resistance leading to public health hazards^65^. Similarly, a high MAR index for *V. parahaemolyticus* isolates was reported previously^66^. The use of nanotechnology to modify their potential effects by regulating their size has been used as a biological alternative control strategy^67–70^. In this study, we evaluated the in vitro antimicrobial efficacy, and transcriptional modulatory effect of AgNPs-H2O2 against MDR microorganisms. The obtained findings showed a notable inhibition zone of AgNPs-H2O2 against *A. hydrophila* strains, *P. aeruginosa* and *Vibrio* species. This observation is supported by a previous study that described a pronounced antibacterial effect of AgNPs against isolated *Vibrio* species^71^. Previous studies also stated an effective MIC of AgNPs against *P. aeruginosa*^37^, *A. hydrophila*, and* Vibrio* species^72,73^. Corroborating these findings, we found that the MIC value of AgNPs-H2O2 against the examined isolates ranged from 0.5 to 8 µg/mL with tolerance level of 2 indicating the bactericidal effect of AgNPs-H2O2. Cytotonic heat-stable enterotoxins (*ast*), cytotoxic enterotoxin (*act*) and aerolysin (*aer A*) genes are the major virulence factor of *A. hydrophila* that exhibit hemolytic and cytolytic properties and causes cell death, depending on destroying the cell membrane permeability causing osmotic lysis^74,75^. Additionally, the *toxA* gene of *P. aeruginosa* inhibits the biosynthesis of host cell proteins^76^. The hemolytic exoenzyme encoded by the *exoS* gene causes tissue destruction and helps in bacterial dissemination^77^, and the exoenzyme-U encoded by the *exoU* gene is considered a marker of *P. aeruginosa* invasiveness as it is the most cytotoxic enzyme among type III secretion proteins^78^. Furthermore, many crucial proteins are commonly used to assess the pathogenicity of Vibrio isolates such as two hemolysins: thermostable direct hemolysin (*tdh*) and the *tdh*-related hemolysin (*trh*)^57,79^, as well as the *toxR* gene which regulates the expression of virulence factors and is used to categorize all isolates as pathogenic strains^80^. Thus, we investigated the transcriptional effect of AgNPs-H2O2 on crucial virulence factors of *A. hydrophila*, *P. aeruginosa*, and *Vibrio* species. This study revealed that *A. hydrophila* isolates treated with AgNPs-H2O2 showed the most significant downregulation of the *aerA* gene by 0.17–0.29-fold change. Additionally, the suppression of *exoU* gene expression in *P. aeruginosa* isolates was more pounced with 0.09–0.21-fold change. Meanwhile, the *trh* gene expression level was the most affected among the tested *Vibrio* species with 0.029–0.21-fold change. This finding is constituent with previous studies that have reported the inhibitory effect of silver nanoparticles on the expression of virulence factors in multidrug-resistant *Pseudomonas aeruginosa* strains^81^. Furthermore, previous research has also documented the significant downregulation of virulence genes in VTEC O157:H7 in response to H2O2^82^.

The antimicrobial activity of the AgNPs-H2O2 (Silver Nanoparticles-Hydrogen Peroxide) combination arises from a synergistic effect between the two components and the breakdown of the mechanisms involved:

1. Silver Nanoparticles (AgNPs) cause direct contact and membrane disruption: AgNPs can physically interact with the bacterial cell wall, causing disruptions and increased permeability. This leakage of essential cellular components leads to cell death. Additionally, AgNPs can interact with oxygen and water molecules within the bacteria, promoting the formation of Reactive Oxygen Species (ROS) like superoxide radicals and hydrogen peroxide. These ROS damage vital cellular components such as proteins, DNA, and lipids^83^.
2. Hydrogen Peroxide (H2O2) induces direct oxidative stress: H2O2 itself can act as a mild oxidizing agent, damaging cellular components to a certain extent^84^.

Thus, the combination of AgNPs and H2O2 can have a synergistic effect on ROS generation. AgNPs can catalyze the decomposition of H2O2 into hydroxyl radicals (OH·), which are even more potent ROS compared to hydrogen peroxide itself. These highly reactive hydroxyl radicals cause extensive damage to cellular components within the bacteria. In addition, H2O2 might also help improve the penetration of AgNPs into the bacterial cells, further enhancing their antimicrobial activity^85^.

Overall, the AgNPs-H2O2 combination disrupts bacterial cell membranes, generates high levels of ROS, leading to oxidative stress and damage to essential cellular components and can be more effective against a broader range of bacteria compared to using AgNPs or H2O2 alone.

This finding could be explained by a higher bactericidal action of more than 100 times due to the Fenton-like reaction between silver nanoparticles and hydrogen peroxide resulting in the formation of hydroxyl groups (–OH) the most potent biologically active ROS^86^. As an alternate antibacterial strategy, this combination is anticipated to provide a number of benefits: (a) the surface chemistry alteration of silver nanoparticles by conjugation of certain moieties, allowing for the selective targeting of bacterial cells and greatly reducing toxicity^87^. (b) Producing silver ions (Ag+), which have antibacterial activity of their own^88^. (c) Silver ions can block the catalase enzyme, which is generated by certain bacteria to neutralize hydrogen peroxide^89^.

In summary, the present study highlights the in vitro inhibitory effect of the dual combination of AgNPs-H2O2 on bacterial growth and the down regulatory effect on the crucial virulence factors of *A. hydrophila, P. aeruginosa,* and *Vibrio* species, making them excellent candidates for targeted drug delivery.

### Supplementary Information

Supplementary Table S1.

Supplementary Table S2.

Supplementary Table S3.

Supplementary Table S4.

Supplementary Legends.

## Data Availability

All data generated or analyzed during this study are included in this published article and its supplementary information files.
