# Supplementary material for: Biocontrol of multidrug resistant pathogens isolated from fish farms using silver nanoparticles combined with hydrogen peroxide insight to its modulatory effect
Source: Sci Rep. 2024 Apr 4;14:7971. doi: 10.1038/s41598-024-58349-4 (PMC10994946; doi:10.1038/s41598-024-58349-4)
Supplement: Supplementary file 1 — Supplementary Table S1. [file 41598_2024_58349_MOESM1_ESM.docx]

Supplementary **Table S1: The utilized oligonucleotide primers in this study**

| Pathogen | Target gene | Primers sequences | Annealing (T°C) | Reference |
| --- | --- | --- | --- | --- |
| *A. hydrophila* | *`16S rRNA* | F;GGGAGTGCCTTCGGGAATCAGA  R; TCACCGCAACATTCTGATTT`G | 55 | ^1^ |
|  | *aerA* | F;CAAGAACAAGTTCAAGTGGCCA  R;ACGAAGGTGTGGTTCCAGT | 55 | ^1^ |
|  | *Act* | F;AGAAGGTGACCACCACCAAGAACA  R; AACTGACATCGGCCTTGAACTC | 55 | ^2^ |
|  | *Ast* | F;TCTCCATGCTTCCCTTCCACT  R;GTGTAGGGATTGAAGAAGCCG | 55 | ^3^ |
| *P.aeruginosa* | *16S rRNA* | F;GGGGGATCTTCGGACCTCA  R;TCCTTAGAGTGCCCACCCG | 55 | ^4^ |
|  | *toxA* | F;GGT AAC CAG CTC AGC CAC AT  R;TGA TGT CCA GGT CAT GCT TC | 55 | ^5^ |
|  | *exoS* | F;CTT GAA GGG ACT CGA CAA GG  R;TTC AGG TCC GCG TAG TGA AT | 60 | ^6^ |
|  | *exoU* | F;GGG AAT ACT TTC CGG GAA GTT  R;CGA TCT CGC TGC TAA TGT GTT | 57 | ^8^ |
| Vibrio species | *16Sr RNA* | F;CAGGCCTAACACATGCAAGTC  R;GCATCTGAGTGTCAGTATCTGTCC | 50 | ^9^ |
|  | *Tdh* | F;CATCTGTCCCTTTTCCTGC  R;CCAAATACATTTTACTTGG | 50 | ^10^ |
|  | *toxR* | F;GTCTTCTGACGCAATCGTTG  R;ATACGAGTGGTTGCTGTCATG | 50 | ^11^ |
|  | *Trh* | F;TTGGCTTCGATATTTTCAGTATCT  R;CATAACAAACATATGCCCATTTCC | 50 | ^11^ |
|  | *V. parahaemolyticus*  (*flaE*) | F;GCAGCTGATCAAAACGTT GAGT  R;ATTATCGATCGTGCCACTCAC | 57 | ^12^ |
|  | *V. vulnificus*  (*hsp*) | F;GTCTTAAAGCGGTTGCTGC  R;CGCTTCAAGTGCTGGTAGAAG | 57 | ^12^ |
|  | *V. alginolyticus*  (*collagenase*) | F;CGAGTACAGTCACTTGAAAGCC  R;CACAACAGAACTCGCGTTACC | 57 | ^12^ |

**References**

1. Wang, G. *et al.* Detection and characterization of the hemolysin genes in *Aeromonas hydrophila* and *Aeromonas sobria* by multiplex PCR. *J Clin Microbiol* **41**, 1048–1054 (2003).

2. Nawaz, M., Sung, K., Khan, S. A., Khan, A. A. & Steele, R. Biochemical and molecular characterization of tetracycline-resistant *Aeromonas veronii* isolates from catfish. *Appl Environ Microbiol* **72**, 6461–6466 (2006).

3. Sen, K. & Rodgers, M. Distribution of six virulence factors in *Aeromonas* species isolated from US drinking water utilities: a PCR identification. *J Appl Microbiol* **97**, 1077–1086 (2004).

4. Abu-Elala, N. M., Abd-Elsalam, R. M., Marouf, S., Abdelaziz, M. & Moustafa, M. Eutrophication, ammonia intoxication, and infectious diseases: interdisciplinary factors of mass mortalities in cultured Nile tilapia. *J Aquat Anim Health* **28**, 187–198 (2016).

5. Wendt, M., De Silva, B. C. & Heo, G.-J. Research Article Virulence Factors and Antimicrobial Resistance of *Pseudomonas aeruginosa* Isolated from Pet Turtles. *Asian J Anim Vet Adv* **12**, 5–11 (2017).

6. Strateva, T. Microbiological and molecular-genetic investigations on resistance mechanisms and virulence factors in clinical strains of *Pseudomonas aeruginosa*. *Medical Univ of Sofia, Bulgaria* (2008).

7. Mehri, I. *et al.* Molecular identification and assessment of genetic diversity of fluorescent pseudomonads based on different polymerase chain reaction (PCR) methods. (2013).

8. Montieri, S., Suffredini, E., Ciccozzi, M., Croci, L. & others. Phylogenetic and evolutionary analysis of *Vibrio parahaemolyticus* and *Vibrio alginolyticus* isolates based on *toxR* gene sequence. *New Microbiologica* **33**, 359–372 (2010).

9. Mustapha, S., Mustapha, E. M. & Nozha, C. *Vibrio alginolyticus*: an emerging pathogen of foodborne diseases. *International Journal of Science and Technology* **2**, 302–309 (2013).

10. Marlina, R. S. *et al.* Detection of *tdh* and *trh* genes in *Vibrio parahaemolyticus* isolated from Corbicula moltkiana prime in West Sumatera, Indonesia. *Southeast Asian J Trop Med Public Health* **38**, 349–355 (2007).

11. Tarr, C. L. *et al.* Identification of Vibrio isolates by a multiplex PCR assay and *rpoB* sequence determination. *J Clin Microbiol* **45**, 134–140 (2007).
