# Supplementary material for: Biocontrol of multidrug resistant pathogens isolated from fish farms using silver nanoparticles combined with hydrogen peroxide insight to its modulatory effect
Source: Sci Rep. 2024 Apr 4;14:7971. doi: 10.1038/s41598-024-58349-4 (PMC10994946; doi:10.1038/s41598-024-58349-4)
Supplement: Supplementary file 5 — Supplementary Legends. [file 41598_2024_58349_MOESM5_ESM.docx]

**Table S2** **(Excel sheet):** The in vitro antimicrobial susceptibilities of *A. hydrophila* against antimicrobial agents.

**Table S3 (Excel sheet):** The in vitro antimicrobial susceptibilities of *P. aeruginosa* against antimicrobial agents.

**Table S4 (Excel sheet):** The in vitro antimicrobial susceptibilities of *Vibrio species* against antimicrobial agents.
